# Efficacy and safety of probiotics in the treatment of pediatric asthma: a systematic review and meta-analysis

**DOI:** 10.3389/fmed.2025.1730100

**Published:** 2026-01-05

**Authors:** Gang Hu, Ke Liao, Yunfeng Yu, Xinyu Yang, Fan Li, Mengqing Wang

**Affiliations:** 1 Department of Pediatrics, The First Hospital of Hunan University of Chinese Medicine, Changsha, Hunan, China; 2 Department of Pediatrics, People’s Hospital of Ningxiang City, Changsha, Hunan, China; 3 School of Traditional Chinese Medicine, Hunan University of Chinese Medicine, Changsha, Hunan, China

**Keywords:** asthma, meta-analysis, pediatric populations, probiotics, systematic review

## Abstract

**Objective:**

Growing evidence suggests that probiotics may offer therapeutic benefits for asthma, attracting increasing scientific attention. To clarify their effects in pediatric patients with asthma, we conducted a systematic review and meta-analysis using trial sequential analysis (TSA).

**Methods:**

A comprehensive search of five electronic databases was performed for studies published until August 30, 2025. Data on study characteristics, outcomes, and risk of bias were extracted. The meta-analysis was conducted using RevMan 5.3, and TSA was performed using TSA 0.9.5.10 beta. Dichotomous outcomes were expressed as risk ratios, and continuous outcomes as mean difference or standardized mean difference (SMD). Publication bias was assessed using funnel plots and the certainty of the evidence was evaluated using the Grading of Recommendations, Assessment, Development, and Evaluation approach.

**Results:**

Six randomized controlled trials (RCTs) involving 731 pediatric patients with asthma were included. The pooled analysis indicated that probiotics significantly reduced interleukin-4 (IL-4) levels [SMD –0.66, 95% confidence intervals (CI) –1.24 to –0.08, *P* = 0.03] and significantly increased those of interferon-γ (INF-γ) (SMD 1.78, 95% CI 0.13–3.44, *P* = 0.03). However, probiotics did not significantly affect daytime or nighttime asthma symptom scores, forced expiratory volume in 1 s, forced vital capacity, peak expiratory flow, or tumor necrosis factor-α levels (*P* ≥ 0.05). TSA did not confirm the conclusiveness of the findings for IL-4 and INF-γ, indicating insufficient evidence to support their clinical significance. Additionally, the funnel plots suggested potential publication bias for these outcomes. The certainty of the evidence for all outcomes was rated as very low.

**Conclusion:**

Although probiotics showed statistically significant effects on IL-4 and INF-γ, the TSA results revealed that neither outcome crossed the monitoring boundaries, indicating that the evidence remains insufficient. Moreover, the very low certainty of the evidence and the fact that all included RCTs were conducted in China suggest that external validity is limited. In view of these uncertainties, the available evidence does not support the routine use of probiotics as an adjunctive therapy in pediatric asthma, and further large-scale, multicenter, RCTs are warranted.

**Systematic review registration:**

https://www.crd.york.ac.uk/PROSPERO/view/CRD420251174081, identifier CRD420251174081.

## 1 Introduction

Asthma is a heterogeneous disease characterized by chronic airway inflammation, airway hyperresponsiveness, and reversible airflow limitations that typically begins early in life (1). Epidemiological studies indicate that approximately 300 million people worldwide suffer from asthma, with children being the most affected group (2). This condition commonly manifests as recurrent wheezing, shortness of breath, chest tightness, and coughing (3). During severe attacks, patients may exhibit restlessness, orthopnea, shoulder elevation during breathing, the “three-concave sign,” nasal flaring, and cyanosis of the lips. Asthma not only interferes with the daily activities and learning of pediatric patients, but can also be life-threatening in severe cases (4). Inadequate or irregular treatment of these patients may lead to poor symptom control, decline in pulmonary function, and increased risk of developing chronic respiratory diseases in adulthood (5). The long-term goals of managing pediatric patients with asthma are to control symptoms, prevent exacerbations, improve lung function, and minimize adverse drug reactions (6). Current treatment strategies primarily include pharmacological interventions such as β_2_-adrenergic agonists and inhaled corticosteroids, as well as non-pharmacological measures including lifestyle modification and exercise (5, 7). In pediatric patients with asthma and concomitant allergic diseases, antihistamines can be administered when necessary (8). Although the effectiveness of these therapies in terms of symptom relief has notably improved, a subset of pediatric patients with asthma still experience suboptimal responses and remain at risk of severe exacerbations (9). Therefore, it is crucial to explore safe and effective adjunctive therapies to further improve clinical outcomes and quality of life in these patients.

The gut microbiota, composed of bacteria, fungi, and other microorganisms residing in the gastrointestinal tract, plays an essential role in maintaining human health, particularly during early life (10, 11). Increasing evidence highlights its relevance for pediatric patients with asthma through the gut–lung axis, a bidirectional network in which microbial metabolites and immune cells circulate between the intestine and the respiratory tract (12, 13). The gut microbiota modulates airway immune responses via metabolites such as short-chain fatty acids and immune cell trafficking (13, 14). Compared to healthy children, those at risk of asthma have significantly reduced relative abundances of genera such as *Lachnospira*, *Veillonella*, *Faecalibacterium*, and *Roth* (15). Subsequent research demonstrated that the proportions of *Bifidobacterium* and *Megasphaera* are significantly lower in pediatric patients with asthma than in healthy controls (16). This dysbiosis may compromise the integrity of the intestinal epithelial barrier, facilitating the systemic translocation of pro-inflammatory molecules such as lipopolysaccharides and thus exacerbating pulmonary inflammation (17, 18). Additionally, gut microbiota imbalance may disrupt the T helper (Th)1/Th2 ratio as well as that of T regulatory (Treg)/Th17, leading to allergic inflammation and asthma symptoms (19). Collectively, these findings highlight the gut microbiota as a critical regulator of pediatric asthma pathogenesis via the gut-lung axis, and suggest that targeting microbial composition could represent a promising avenue for preventive or therapeutic strategies.

However, previous studies have reported conflicting findings regarding whether probiotics can improve the prognosis of pediatric patients with asthma. A meta-analysis by Xie et al. (20) reported that probiotics significantly reduced fractional exhaled nitric oxide and the severity of asthma symptoms, and increased the Childhood Asthma Control Test (CACT) score, although no effects were observed on the forced expiratory volume in 1 s (FEV_1_) or on the FEV_1_/forced vital capacity (FVC) ratio. Another meta-analysis by Lin et al. (21) found that probiotics reduced the frequency of asthma exacerbation, decreased interleukin-4 (IL-4) levels, and increased those of interferon-γ (IFN-γ), but did not significantly affect CACT scores, daytime or nighttime asthma symptoms, FEV_1_, or peak expiratory flow (PEF). Similarly, Liu et al. (22) reported that probiotics significantly reduced the risk of acute asthma attacks and improved FEV_1_/FVC but had no significant effect on FEV_1_. These inconsistencies reveal an ongoing controversy regarding the effects of probiotics on various clinical symptoms, inflammatory responses, and pulmonary function outcomes in pediatric patients with asthma.

Notably, the studies by Xie et al. (20) and Lin et al. (21) included pediatric patients with recurrent wheezing and a first-degree family history of atopic diseases. The heterogeneity of the study populations may have introduced additional confounding factors and reduced the precision of the pooled estimates. Moreover, Xie et al. (20) and Liu et al. (22) included non-randomized controlled trials, which further increased methodological heterogeneity and undermined the reliability and precision of their findings. To address these limitations, we conducted a more rigorous meta-analysis restricted to randomized controlled trials (RCTs) involving pediatric patients with a confirmed diagnosis of asthma, and incorporating trial sequential analysis (TSA). Individuals with recurrent wheezing and other high-risk populations were excluded from the study, effectively reducing participant-related clinical heterogeneity. In addition, unlike previous meta-analyses, the present study is the result of a comprehensive search in both international and Chinese databases, with the Chinese literature restricted to the Chinese Science Citation Database (CSCD) to enhance the rigor of the evidence base. Furthermore, compared with previous studies, the present study incorporated additional inflammation markers such as tumor necrosis factor-α (TNF-α) and other cytokines, as well as lung function parameters including FVC, to more comprehensively assess the potential effects of probiotics on asthma-related symptoms, inflammatory responses, and pulmonary function in pediatric patients with asthma. By adopting these measures for the study, we aimed to provide more robust and precise evidence regarding the potential role of probiotics as an adjunctive therapy for pediatric patients with asthma.

## 2 Methods

This meta-analysis was conducted in accordance with the Preferred Reporting Items for Systematic Reviews and Meta-Analyses (PRISMA) guidelines, and was registered in the PROSPERO database (CRD420251174081). The primary objective of this study was to evaluate the effects of probiotics in pediatric patients with asthma.

### 2.1 Inclusion and exclusion criteria

Inclusion criteria: (i) Participants: Pediatric patients aged 0–18 years diagnosed with asthma, without restrictions on sex, ethnicity, or disease duration. (ii) Interventions: Probiotics supplementation administered either alone or in combination with standard pharmacological therapy for asthma. (iii) Comparisons: Standard pharmacological therapy for asthma alone or placebo. (iv) Outcomes: asthma-related outcomes including scores for daytime and nighttime symptoms; Pulmonary function outcomes including FEV_1_, FVC and PEF; Inflammatory biomarkers including TNF-α, IL-4 and IFN-γ. (v) Study design: RCTs only.

The exclusion criteria were as follows: (i) Duplicate publications or retracted articles; (ii) Publications with full text unavailable or with incomplete data; and (iii) Publications containing evident statistical errors.

### 2.2 Literature search strategy

A comprehensive literature search was performed across five electronic databases: The CSCD, PubMed, Embase, Web of Science, and ClinicalTrials.gov. The first four databases were used to identify published studies, whereas ClinicalTrials.gov was searched for gray literature. The search strategy combined Medical Subject Headings (MeSH) and free-text terms in the title or abstract, including: [(Asthma OR Asthmas) AND (Probiotic OR Probiotics OR Bifidobacterium OR Bifidobacteria OR *Bacillus bifida* OR Yeast OR *Saccharomyces cerevisiae* OR *Saccharomyces italicus* OR *Saccharomyces oviformis* OR *S cerevisiae* OR *S. cerevisiae* OR *Saccharomyces uvarum var melibiosus* OR *Candida robusta* OR *Saccharomyces capensis* OR *Lactobacillus acidophilus* OR *Lactobacillus amylovorus* OR Lactobacill OR Lactic acid bacteria OR *Clostridium butyricum* OR Bacillus OR Natto Bacteria OR *Streptococcus thermophiles* OR *Enterococcus*)]. The search covered all available records from the inception of each database to August 30, 2025, without language restrictions. Additionally, the reference lists of relevant reviews and included studies were manually screened to ensure comprehensive coverage. The search strategy and results for each database are shown in Supplementary Table 1.

### 2.3 Study selection process

All retrieved records were imported into Endnote X9 software, where duplicates were initially removed automatically and subsequently verified manually to enhance methodological rigor. Two independent reviewers then systematically screened the titles and abstracts in strict accordance with the predefined inclusion and exclusion criteria. The full-text version of all articles considered as potentially eligible were obtained and thoroughly assessed by both reviewers. Any discrepancies were resolved through discussion, and when a consensus could not be reached, a third reviewer was consulted to ensure objectivity. The entire study selection process was comprehensively documented and presented in a visual manner through the PRISMA flow diagram.

### 2.4 Data extraction

Data were extracted independently by two reviewers using a standardized form. The information retrieved included authorship, publication year, sample size, participant demographics, intervention and control characteristics. To prevent bias from duplicate or overlapping cohorts, all included studies were carefully cross-checked for similarities in research groups, study sites, and recruitment periods. When overlapping datasets were identified, only the study with the most complete data, longest follow-up, or largest sample size was retained to ensure independence of the observations. For outcomes with missing or incomplete data, appropriate statistical procedures were applied. When standard deviations were not explicitly reported, they were derived from standard errors, confidence intervals, or interquartile ranges, in accordance with the Cochrane Handbook. These steps ensured consistency in data extraction and minimized the risk of bias related to duplicate datasets or incomplete reporting.

### 2.5 Risk of bias assessment

The methodological quality of the included trials was independently evaluated by two reviewers using the Cochrane Risk of Bias tool, covering sequence generation, allocation concealment, blinding procedures, incomplete outcome data, selective reporting, and other potential sources of bias. Domains were rated as low, high, or unclear risk, with an overall study-level judgment. Discrepancies were resolved through consensus or third-party adjudication.

### 2.6 Data synthesis and statistical analysis

Meta-analyses were performed using the Review Manager software (version 5.3). Risk ratios (RR) with 95% confidence intervals (CI) were calculated for dichotomous outcomes, whereas the mean difference (MD) or standardized mean difference (SMD) with 95% CI was used for continuous outcomes, depending on the measurement tools. Heterogeneity was assessed with Cochran’s *Q*-test and the *I*^2^ statistic, with *P* ≥ 0.10 and *I*^2^ < 50% indicating low heterogeneity; otherwise, heterogeneity was considered high. Fixed-effects models were applied when heterogeneity was low, and random-effects models were used in the remaining cases. In cases in which substantial heterogeneity was detected for a given outcome and more than three studies were available, sensitivity and exploratory subgroup analyses were conducted to identify potential sources of heterogeneity. Subgroup analyses were conducted based on key clinical variables including average age, disease duration, treatment duration, probiotic type, and probiotic dosage.

Additionally, TSA was performed using the TSA software (version 0.9.5.10 beta) to control for random errors and assess the reliability of cumulative evidence. The required information size was calculated based on the anticipated effect size estimated from the observed MD. The MD and standard deviation were automatically computed by the software from the extracted data, with a two-sided type I error of 5% and a power of 80%. TSA monitoring boundaries were applied to determine whether the cumulative evidence was sufficient for definitive conclusions; crossing the boundary indicated conclusive results, whereas failure to cross suggested the need for further studies.

### 2.7 Publication bias

Publication bias was assessed using funnel plots, in which the MD or SMD were plotted on the *x*-axis against their standard errors on the *y*-axis. The distribution symmetry of the plotted studies was visually inspected to provide an initial indication of potential publication bias or small-study effects. When the number of included studies was ≥ 10, the Egger test was applied to quantitatively assess publication bias; otherwise, visual inspection of the funnel plot was used as the primary evaluation method.

### 2.8 Certainty of evidence

The certainty of evidence for each outcome was assessed using the Grading of Recommendations, Assessment, Development, and Evaluation (GRADE) system, which accounts for the risk of bias, inconsistency, indirectness, imprecision, and publication bias. Outcomes were categorized as high, moderate, low, or very low quality of evidence, guiding confidence in the pooled estimates and subsequent recommendations.

## 3 Results

### 3.1 Study selection

**FIGURE 1:**
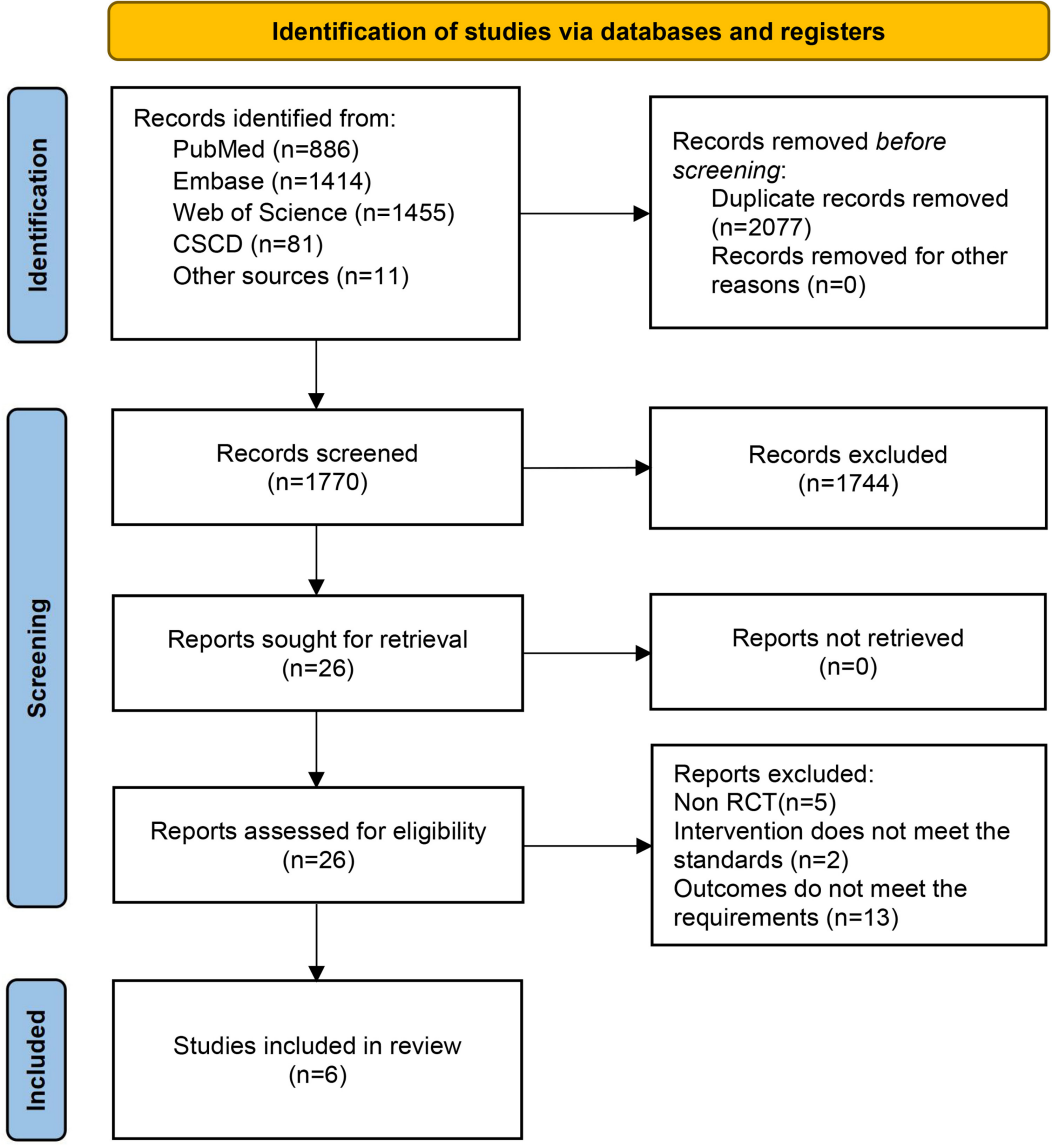
Literature screening process. CSCD, Chinese Science Citation Database.

The comprehensive literature search across various databases yielded a total of 3,847 records: 886 from PubMed, 1,414 from Embase, 1,455 from Web of Science, 81 from CSCD, and 11 from other sources. After eliminating 2,077 duplicate entries, 1,770 records were retained for screening based on the titles and abstracts. This initial screening led to the exclusion of 1,744 studies that were deemed irrelevant. The full texts of the remaining articles were then evaluated for eligibility, resulting in the exclusion of 20 studies: Five were excluded because they were not RCTs, two did not meet the intervention standards, and 13 failed to meet the outcome requirements. Ultimately, six studies were included in this meta-analysis (23–28). A detailed flow diagram is presented in Figure 1.

### 3.2 Basic characteristics of included studies

The meta-analysis included six RCTs with a total of 731 participants (23–28). These studies were published between 2010 and 2022. All studies were conducted in China. The male ratio ranged from 48.91% to 60.87%. The mean age ranged from 6.36 to 11.2 years. Two studies utilized probiotics formulations containing a single strain, whereas four used multi-strain preparations. The treatment duration varied from 2 to 12 weeks. Additional characteristics of the included studies are summarized in Table 1.

**TABLE 1:** Basic characteristics of included studies.

Author name	Sample size	Age (years)	Male (%)	Disease duration (months)	Intervention	Treatment duration (weeks)
Ding R 2022 (27)	75	6.36 ± 4.54	58.67%	/	6.0 × 10^6^ CFU/d *Bifidobacterium* spp., 6.0 × 10^6^ CFU/d *Lactobacillus acidophilus*, 6.0 × 10^6^ CFU/d *Enterococcus faecium*, 6.0 × 10^5^ CFU/d *Bacillus cereus* + Budesonide suspension 0.25 mg Bid	2
	75	6.89 ± 5.12	52.00%	/	Budesonide suspension, 0.25 mg Bid	
Zhang PH 2017 (26)	94	7.0 ± 3.3	60.64%	18.4 ± 4.5	2.6 × 10^9^ CFU/d *Saccharomyces boulardii* CNCM I-745 + GINA regimen treatment	4
	90	6.8 ± 3.9	61.11%	18.0 ± 4.9	GINA regimen treatment	
Xia QX 2022 (25)	46	6.84 ± 2.71	52.17%	28.62 ± 9.34	2.0 × 10^7^ CFU/d *Bifidobacterium longum*, *Lactobacillus acidophilus* and *Enterococcus faecalis* + Budesonide suspension, 1 mg Bid + Terbutaline sulfate solution for nebulized inhalation, > 20 kg 5 mg/ < 20 kg 2.5 mg Bid + Ipratropium bromide solution for inhalation, 250 μg Bid. After symptom relief, switch to Budesonide suspension 0.5 mg Bid	4
	46	6.53 ± 2.59	45.65%	28.21 ± 9.20	Budesonide suspension, 1 mg Bid + Terbutaline sulfate solution for nebulized inhalation, > 20 kg 5 mg/ < 20 kg 2.5 mg Bid + Ipratropium bromide solution for inhalation, 250 μg Bid. After symptom relief, switch to Budesonide suspension 0.5 mg Bid	
Zhen XG 2018 (24)	27	11.2 ± 1.8	55.56%	/	2.0 × 10^7^ CFU/d *Bifidobacterium longum*, *Lactobacillus acidophilus* and *Enterococcus faecalis* + Budesonide suspension 1.0 mg Bid	6
	26	10.9 ± 1.5	50.00%	/	Budesonide suspension 1.0 mg Bid	
Huang CF 2018 (28)	38	7.68 ± 2.21	57.89%	/	2.0 × 10^9^CFU/d *Lactobacillus paracasei* GMNL-133	12
	38	7.37 ± 2.34	63.16%	/	2.0 × 10^9^ CFU/d *Lactobacillus fermentum* GM-090	12
	36	7.00 ± 1.79	52.78%	/	2.0 × 10^9^CFU/d *Lactobacillus paracasei* GMNL-133 + 2.0 × 10^9^ CFU/d *Lactobacillus fermentum* GM-090	12
	35	7.86 ± 2.50	51.43%	/	Placebo Qd	
Chen YS 2010 (23)	49	8.1 ± 3.0	57.10%	/	4 × 10^9^ CFU/d *Lactobacillus gasseri* PM-A0005	8
	56	9.4 ± 4.1	57.10%	/	Placebo Bid	

### 3.3 Risk of bias

**FIGURE 2:**
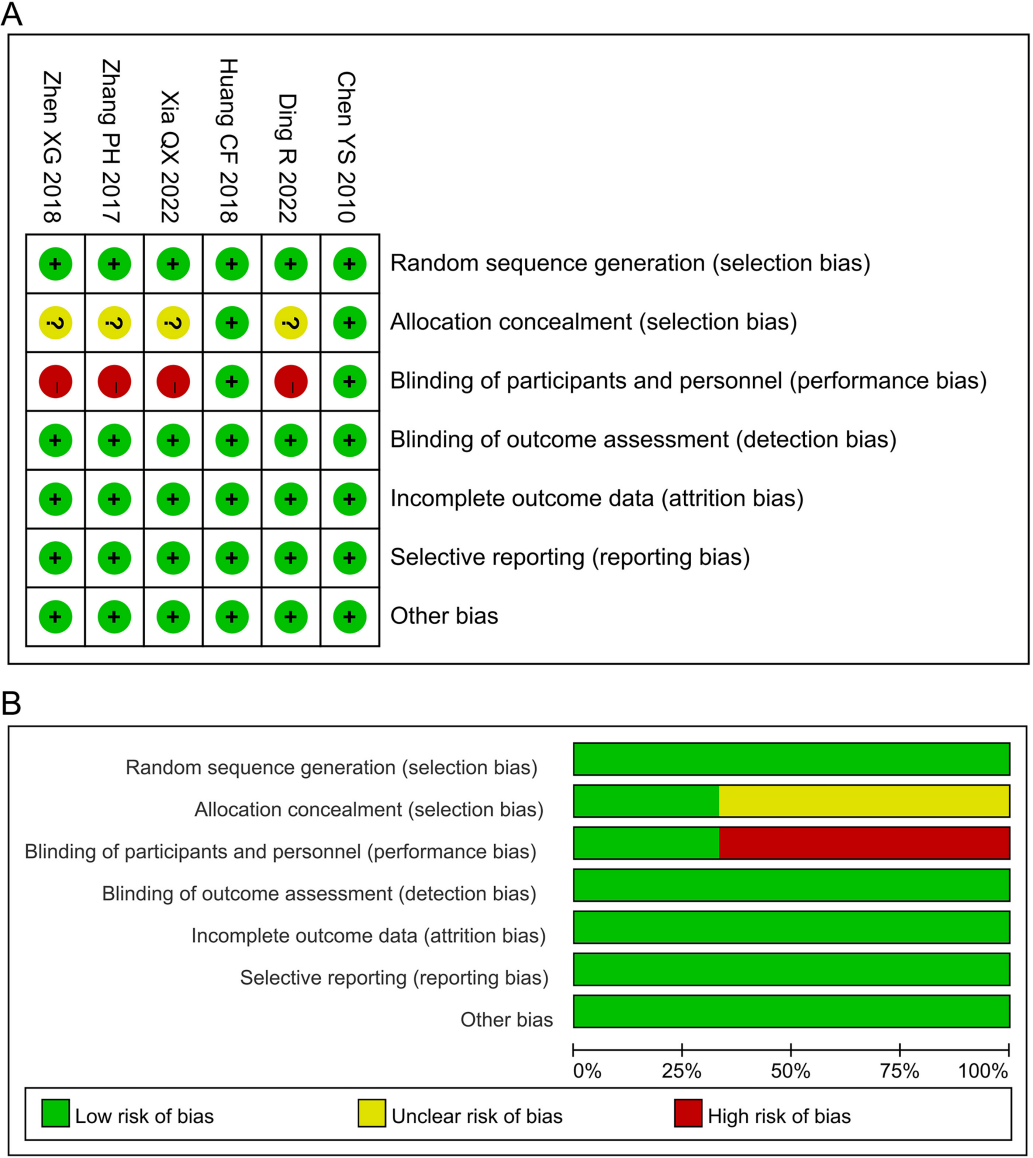
Risk assessment of bias. **(A)** Risk of bias summary; **(B)** risk of bias graph.

All six included trials adopted computer-generated random sequence, indicating a low risk of bias in the random sequence generation. Only two studies (23, 28) explicitly reported allocation concealment and placebo-controlled design, and were therefore judged to be at low risk for allocation concealment and blinding of participants and personnel. As the primary outcomes were assessed using objective measures, the risk of bias from the outcome assessment was also low. The attrition rates were below 20% in all studies, suggesting a low risk of attrition bias. Furthermore, all trials had pre-specified outcomes, with no evidence of selective reporting or other biases. Collectively, the risk of bias across all domains was low (Figure 2).

### 3.4 Meta-analysis

#### 3.4.1 Asthma-related symptoms

##### 3.4.1.1 Asthma daytime symptom score

**FIGURE 3:**
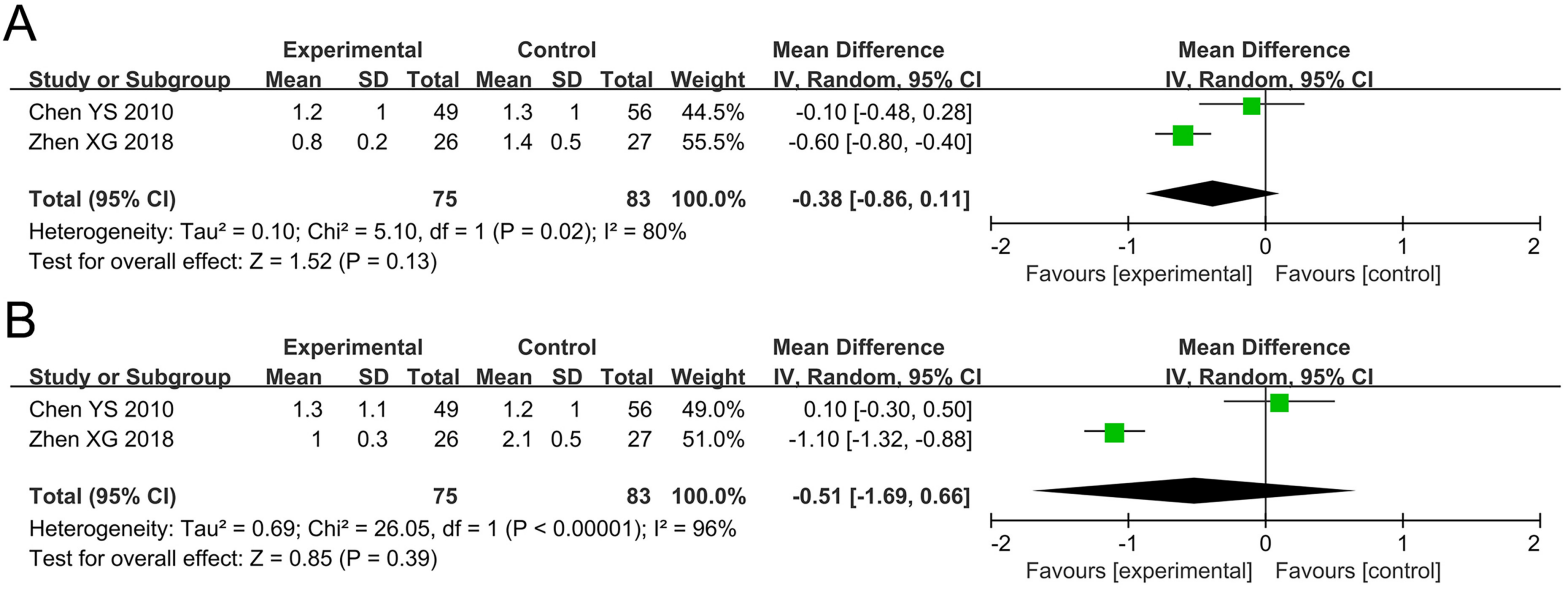
Forest plots of the meta-analysis on asthma-related symptoms: **(A)** Asthma daytime symptom score; **(B)** asthma nighttime symptom score. CI, confidence interval; IV, inverse variance; MD, mean difference; SD, standard deviation. Each horizontal bar represents an individual study, with the size of the square in the middle of the bar indicating the study weight. The length of the horizontal bar corresponds to the study’s CI, the solid line denotes the vertical line at the null value, and the diamond symbol indicates the pooled effect size.

The meta-analysis for asthma daytime symptom score included two studies involving 158 participants. Cochran’s *Q*-test and the *I*^2^ statistic indicated high heterogeneity (*P* = 0.02; *I*^2^ = 80%). The pooled results indicated no statistically significant difference in asthma daytime symptom score between the probiotics and non-probiotics groups (MD –0.38, 95% CI –0.86 to 0.11, *P* = 0.13; Figure 3A).

##### 3.4.1.2 Asthma nighttime symptom score

The meta-analysis for asthma nighttime symptom score included two studies involving 158 participants. The Cochran’s *Q*-test and the I^2^ statistic indicated high heterogeneity (*P* < 0.00001; *I*^2^ = 96%). The pooled results indicated no statistically significant difference in asthma nighttime symptom score between the probiotics and non-probiotics groups (MD –0.51, 95% CI –1.69 to 0.66, *P* = 0.39; Figure 3B).

#### 3.4.2 Pulmonary function

##### 3.4.2.1 FEV_1_

**FIGURE 4:**
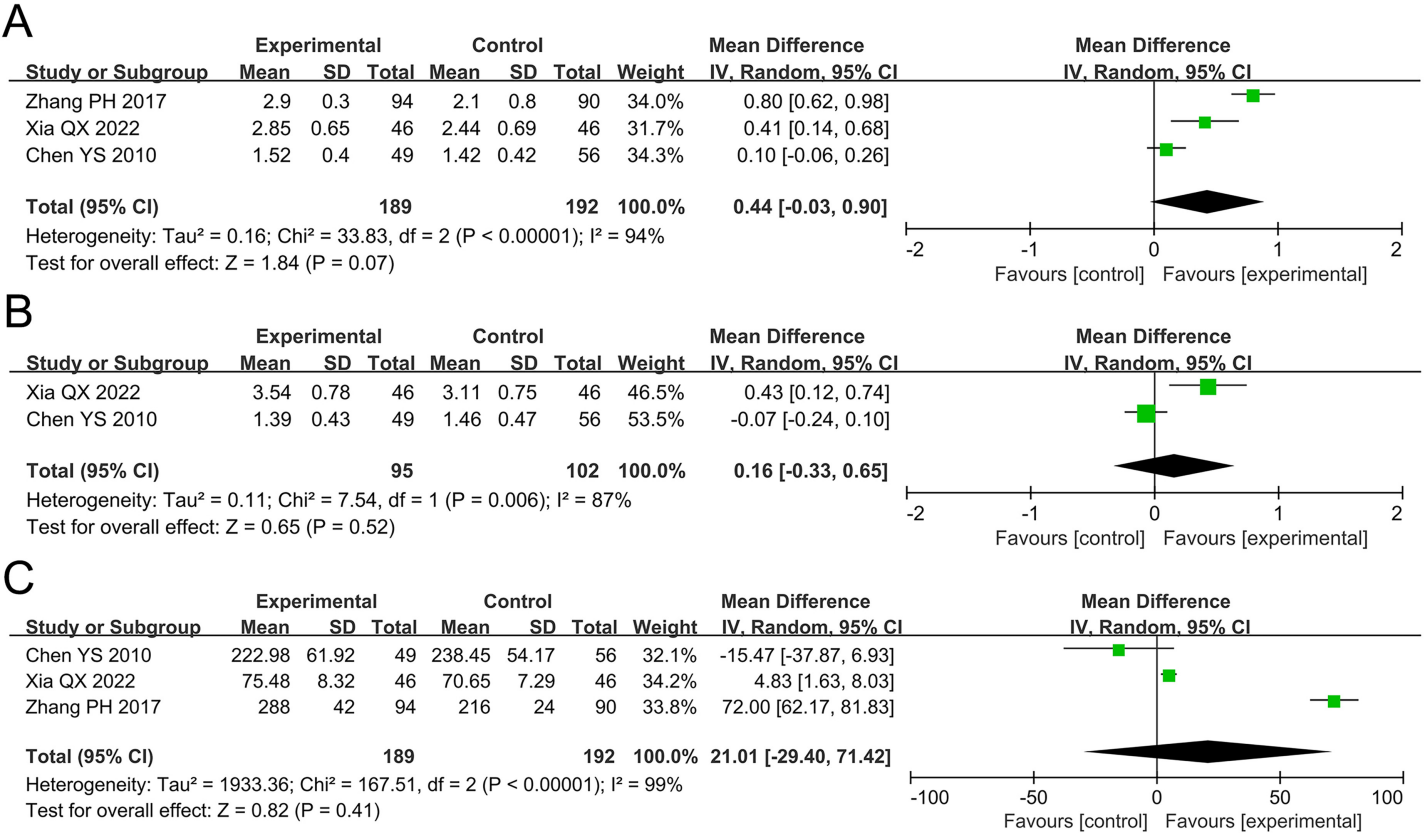
Forest plots of the meta-analysis on pulmonary function: **(A)** FEV_1_; **(B)** FVC; **(C)** PEF. FEV_1_, forced expiratory volume in one second; FVC, forced vital capacity; PEF, peak expiratory flow; CI, confidence interval; IV, inverse variance; MD, mean difference; SD, standard deviation. Each horizontal bar represents an individual study, with the size of the square in the middle of the bar indicating the study weight. The length of the horizontal bar corresponds to the study’s CI, the solid line denotes the vertical line at the null value, and the diamond symbol indicates the pooled effect size.

The meta-analysis for FEV_1_ included three studies involving 381 participants. Cochran’s *Q*-test and the *I*^2^ statistic indicated high heterogeneity (*P* < 0.00001; *I*^2^ = 94%). The pooled results demonstrated no statistically significant difference in FEV_1_ between the probiotics and non-probiotics groups (MD 0.44 L, 95% CI –0.03 to 0.90, *P* = 0.07; Figure 4A).

##### 3.4.2.2 FVC

The meta-analysis for FVC included two studies involving 197 participants. Cochran’s *Q*-test and the *I*^2^ statistic indicated high heterogeneity (*P* = 0.006; *I*^2^ = 87%). The pooled results revealed no statistically significant difference in FVC between the two groups (MD 0.16 L, 95% CI –0.33 to 0.65, *P* = 0.52; Figure 4B).

##### 3.4.2.3 PEF

The meta-analysis for PEF included three studies involving 381 participants. Cochran’s *Q*-test and the *I*^2^ statistic indicated high heterogeneity (*P* < 0.00001; *I*^2^ = 99%). The pooled results revealed no statistically significant difference in PEF between the two groups (MD 21.01 L/min, 95% CI –29.40 to 71.42, *P* = 0.41; Figure 4C).

#### 3.4.3 Inflammatory biomarkers

##### 3.4.3.1 TNF-α

**FIGURE 5:**
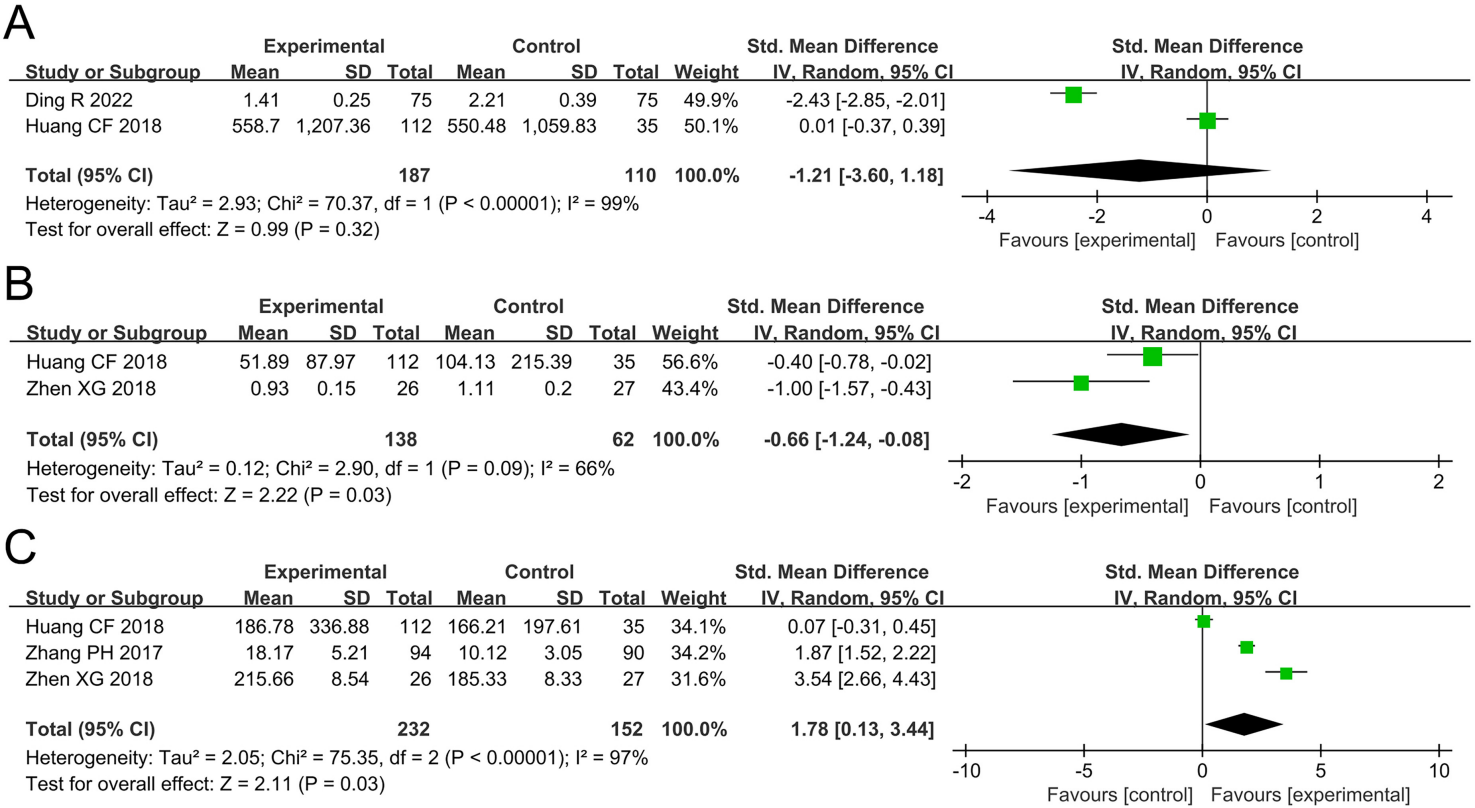
Forest plots of the meta-analysis on inflammatory biomarkers: **(A)** TNF-α; **(B)** IL-4; **(C)** IFN-γ. TNF-α, tumor necrosis factor-alpha; IL-4, interleukin-4; IFN-γ, interferon-gamma; CI, confidence interval; IV, inverse variance; SMD, standardized mean difference; SD, standard deviation. Each horizontal bar represents an individual study, with the size of the square in the middle of the bar indicating the study weight. The length of the horizontal bar corresponds to the study’s CI, the solid line denotes the vertical line at the null value, and the diamond symbol indicates the pooled effect size.

The meta-analysis for TNF-α levels included two studies involving 297 participants. Cochran’s *Q*-test and the *I*^2^ statistic indicated high heterogeneity (*P* < 0.00001; *I*^2^ = 99%). The pooled results revealed no statistically significant difference in TNF-α levels between the two groups (SMD –1.21, 95% CI –3.60 to 1.18, *P* = 0.32; Figure 5A).

##### 3.4.3.2 IL-4

The meta-analysis for IL-4 levels included two studies involving 200 participants. Cochran’s *Q*-test and the I^2^ statistic indicated high heterogeneity (*P* = 0.09; *I*^2^ = 66%). The pooled results demonstrated a significant reduction in IL-4 levels in the probiotics group compared to the non-probiotics group (SMD –0.66, 95% CI –1.24 to –0.08, *P* = 0.03; Figure 5B).

##### 3.4.3.3 IFN-γ

The meta-analysis for IFN-γ levels included three studies involving 384 participants. Cochran’s *Q*-test and the *I*^2^ statistic indicated high heterogeneity (*P* < 0.00001; *I*^2^ = 97%). The pooled results revealed a significant increase in IFN-γ levels in the probiotics group compared to the non-probiotics group (SMD 1.78, 95% CI 0.13–3.44, *P* = 0.03; Figure 5C).

#### 3.4.4 Safety outcomes

Among the included studies, only two studies (23, 27) reported safety outcomes, with both indicating that no adverse events related to probiotics use occurred during the study period. However, the current evidence is insufficient to conclusively establish the safety of probiotics in pediatric patients with asthma.

### 3.5 TSA

**FIGURE 6:**
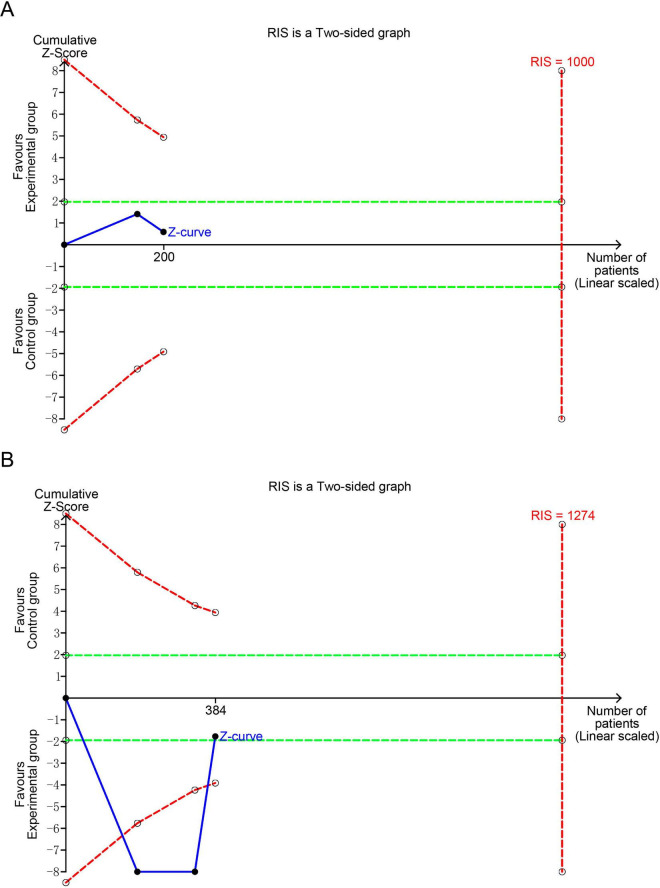
Trial sequential analyses of positive outcomes: **(A)** IL-4; **(B)** IFN-γ. IL-4, interleukin-4; IFN-γ, interferon-γ; RIS, required information size. The blue curve represents the Z-curve, while the red curves above and below represent the trial sequential monitoring boundaries, the dashed green line represents the conventional level of statistical significance, the red vertical line represents the required information size, and the solid dot represents the included study. If the Z-curve does not cross the RIS boundary, it indicates that the outcome lacks conclusive evidence.

TSA was conducted to evaluate the reliability and validity of the meta-analysis results for IL-4 and IFN-γ, and to minimize the risk of false positives, as illustrated in Figures 6A,B. The analysis was performed using a two-sided type I error of 5% and a statistical power of 80%. The anticipated effect sizes were estimated based on the observed MD (–12.91 for IL-4 and 19.15 for IFN-γ). TSA calculated the required information size (RIS) (1,000 for IL-4 and 1,274 for IFN-γ) and generated cumulative Z-curves. The analysis revealed that the *Z*-value curve for IL-4 and IFN-γ did not cross the monitoring boundaries. This implies that the result for IL-4 and IFN-γ needs further verification through additional studies with a similar design.

### 3.6 Publication bias

**FIGURE 7:**
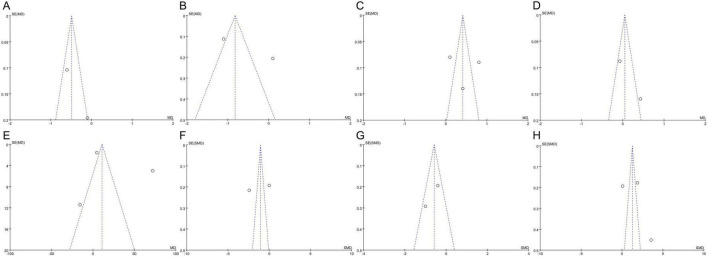
Funnel plots of publication bias: **(A)** Asthma daytime symptom score; **(B)** asthma nighttime symptom score; **(C)** FEV_1_; **(D)** FVC; **(E)** PEF; **(F)** TNF-α; **(G)** IL-4; **(H)** IFN-γ. FEV_1_, forced expiratory volume in 1 s; FVC, forced vital capacity; PEF, peak expiratory flow; TNF-α, tumor necrosis factor-alpha; IL-4, interleukin-4; IFN-γ, interferon-gamma; MD, mean difference; SMD, standardized mean difference; SE, standard error. The circles represent the effect size and standard error of each individual study, while the central vertical dashed line indicates the pooled effect estimate of the meta-analysis, and the two diagonal dashed lines denote the 95% confidence limits.

Publication bias was examined using funnel plots for asthma daytime and nighttime symptom scores, FEV_1_, FVC, PEF, and the levels of TNF-α, IL-4, and IFN-γ (Figure 7). The analysis revealed no significant publication bias for the nighttime symptom score, FEV_1_, PEF, and TNF-α levels, while there was publication bias for the daytime symptom score, FVC, and the levels of IL-4, and IFN-γ. However, the Egger test to assess publication bias was not conducted owing to the insufficient number of included studies, as fewer than 10 were available for analysis. The effectiveness of Egger’s test is significantly reduced with such a small sample size, which may lead to unreliable results.

### 3.7 Certainty of evidence

According to the GRADE system, the certainty of evidence for the asthma daytime and nighttime symptom scores, FEV_1_, FVC, PEF, and the levels of TNF-α, IFN-γ, and IL-4 was classified as very low, underscoring the need for additional rigorously designed studies to validate these findings (Table 2).

**TABLE 2:** Certainty of evidence.

Outcome	Risk of bias	Inconsistency	Indirectness	Imprecision	Publication bias	MD (95% CI)	Certainty of evidence
Asthma daytime symptom score	Serious^(a)^	Serious^(b)^	None	Serious^(c)^	Suspected^(d)^	–0.38 (–0.86, 0.11)	Very Low
Asthma nighttime symptom score	Serious^(a)^	Serious^(b)^	None	Serious^(c)^	None	–0.51 (–1.69, 0.66)	Very low
FEV_1_	Serious^(a)^	Serious^(b)^	None	Serious^(c)^	None	0.44 (–0.03, 0.90)	Very low
FVC	Serious^(a)^	Serious^(b)^	None	Serious^(c)^	Suspected^(d)^	0.16 (–0.33, 0.65)	Very low
PEF	Serious^(a)^	Serious^(b)^	None	Serious^(c)^	None	21.01 (–29.40, 71.42)	Very low
TNF-α	Serious^(a)^	Serious^(b)^	None	Serious^(c)^	None	–1.21 (–3.60, 1.18)	Very low
IL-4	Serious^(a)^	Serious^(b)^	None	Serious^(c)^	Suspected^(d)^	–0.66 (–1.24, –0.08)	Very low
IFN-γ	Serious^(a)^	Serious^(b)^	None	Serious^(c)^	Suspected^(*d*)^	1.78 (0.13, 3.44)	Very low

MD, mean difference; CI, confidence interval; FEV_1_, forced expiratory volume in 1 s; FVC, forced vital capacity; PEF, peak expiratory flow; TNF-α, tumor necrosis factor-alpha; IL-4, interleukin-4; IFN-γ, interferon-gamma. ^(a)^At least one included study has a potential risk of bias. ^(b)^There is high or extremely high heterogeneity. ^(c)^The broad confidence interval decreases credibility. ^(d)^The funnel plot suggests potential publication bias.

## 4 Discussion

Compared to previous meta-analyses, our study provides new insights and methodological improvements. Unlike earlier analyses, we excluded studies involving pediatric patients with recurrent wheezing and a first-degree family history of atopic disease to minimize potential confounding factors. This strategy allowed us to more accurately assess the true effects of probiotics on asthma rather than merely on wheezing episodes. Our findings indicated that probiotic supplementation significantly reduced IL-4 levels and increased those of IFN-γ in pediatric patients with asthma. However, no significant effects were found on asthma daytime or nighttime symptom scores, FEV1, FVC, PEF, or TNF-α levels. Notably, TSA results indicated that these findings were inconclusive, implying that the true benefits of probiotics remain uncertain. This uncertainty is further reflected in the overall quality of evidence for the efficacy of probiotics in asthma treatment, which was classified as “very low.” Consequently, the current data do not provide sufficient support for the widespread use of probiotics as an adjunctive treatment for asthma. Clinicians should exercise caution when considering probiotics for asthma management because their clinical benefits remains uncertain.

Regarding asthma-related symptoms, no significant differences were observed between the probiotics and the control groups in terms of daytime or nighttime asthma symptom scores. Interestingly, two of the included studies (23, 24) reported significant reductions in both daytime and nighttime symptom scores in the probiotics group compared to the control group. We speculate that this inconsistency may stem from both clinical and statistical heterogeneity. First, the two studies reporting asthma-related outcomes differed in terms of participants and interventions. Zhen et al. (24) included pediatric patients with asthma aged 1–14 years, among whom only 54.71% had a history of allergic diseases, while Chen et al. (23) enrolled pediatric patients with asthma aged 6–12 years with mild-to-moderate persistent asthma lasting longer than a year, all of whom had concomitant allergic rhinitis. Zhen et al. (24) used a multi-strain probiotics preparation containing a combination of live *Bifidobacterium*, *Lactobacillus*, and *Enterococcus*, whereas Chen et al. (23) used a single-strain formulation containing *Lactobacillus gasseri* PM-A0005. Second, when the analysis model was switched to a fixed-effect model, significant differences emerged for both daytime (MD –0.49, 95% CI –0.67 to –0.31, *P* < 0.00001) and nighttime symptom scores (MD –0.82, 95% CI –1.02 to –0.63, *P* < 0.00001). However, despite the statistical significance, the effect sizes were small (0.49 and 0.82, respectively), suggesting that probiotics have negligible clinical benefits on asthma-related symptoms. Additionally, Kardani et al. (29) reported that probiotics combined with immunotherapy showed no significant improvement in the Asthma Control Test score compared to immunotherapy alone, further supporting the ineffectiveness of probiotics in improving asthma-related symptoms. Collectively, the available evidence indicates that probiotics offer minimal, if any, benefits in the alleviation of asthma symptoms in pediatric patients.

Regarding pulmonary function, pediatric patients with asthma typically exhibit obstructive ventilatory dysfunction characterized by decreased FEV_1_, FEV_1_/FVC, and PEF values (30). Pulmonary function not only reflects the degree of control over asthma symptoms but also correlates closely with prognosis (31). The results of this meta-analysis showed no significant differences in FEV_1_, FVC, or PEF between the probiotics and control groups. Similar findings were reported by Xie et al. (20), who observed no improvement in FEV_1_, and by Lin et al. (21), who found no significant benefit in either FEV_1_ or PEF. However, Liu et al. (32) found that although probiotics did not significantly affect FEV_1_, they markedly increased the FEV_1_/FVC ratio. These conflicting findings may result from additional confounding factors. Specifically, both Xie et al. (20) and Lin et al. (21) included a study by Rose et al. (33) involving pediatric patients with recurrent wheezing and a first-degree family history of atopic diseases, while Xie et al. (20) and Liu et al. (22) included in the analysis a non-randomized controlled trial reported by Moura et al. (34). The inclusion of participants with recurrent wheezing and of studies with non-randomized designs may have introduced confounding effects and biased the pooled results. In contrast, we excluded these studies from our analysis, thereby reducing potential confounding factors. The results suggested that probiotics exert no significant impact on pulmonary function in pediatric patients with asthma. Moreover, Chen et al. (23) found no significant difference in the change of MEF 25–75% between the probiotics and control groups (*P* = 0.211), indicating that probiotics do not appear to improve small airway function in pediatric patients with asthma. Collectively, these findings suggest that probiotics have no meaningful clinical effect on lung function in pediatric asthma. However, due to the limited sample size and short duration of the intervention in the included studies, the impact of probiotics on pulmonary function remains uncertain and warrants further investigation through large-scale RCTs.

TNF-α plays a critical role in the regulation of inflammatory responses and induction of airway hyperresponsiveness (35, 36). IL-4, a key driver of Th2-type inflammation, is central to airway inflammation and remodeling in asthma (37, 38). In contrast, IFN-γ serves as a hallmark cytokine of Th1-type immune responses and can suppress asthma-related inflammation (39). Our meta-analysis demonstrated that probiotics significantly reduced IL-4 levels (SMD –0.66, 95% CI –1.24 to –0.08) and increased those of IFN-γ (SMD 1.78, 95% CI 0.13–3.44), while showing no significant effect on TNF-α levels. These findings are consistent with those reported by Lin et al. (21), who also observed that probiotics decreased IL-4 levels and increased IFN-γ levels in pediatric patients with asthma. From a mechanistic perspective, these results suggest that probiotics may help re-balance immune function in pediatric asthma. The reduction in IL-4 levels indicates suppression of Th2-mediated allergic inflammation, while the increase in IFN-γ levels reflects potential enhancement of Th1-type responses. In terms of effect size, the IL-4 reduction represents a moderate effect, whereas the increase in IFN-γ levels corresponds to a large effect. Together, these changes imply that probiotics can help rebalance immune function by suppressing Th2-type responses and enhancing Th1-type responses in pediatric patients with asthma. Notably, although the variations in IL-4 and IFN-γ levels showed statistical significance in the pooled analysis, the SMD reflects a standardized statistical effect rather than a clinically meaningful one. The wide confidence intervals and high heterogeneity further complicate the interpretation of these results. Taken together, while probiotics may show potential for immune modulation, current evidence does not support their widespread clinical application in pediatric asthma. The clinical efficacy of probiotics, particularly on the regulation of inflammatory cytokine levels, remains unclear.

The limited efficacy of probiotics can be attributed to strain-specific characteristics and host-related immunological barriers. Probiotics exhibit strain-dependent survival and persistence in the gastrointestinal tract (40). During mucosal inflammation, disrupted tight junctions and increased antimicrobial peptide secretion create a hostile environment that impairs probiotic adhesion and survival, thereby reducing their ability to modulate immune responses (41). Probiotics are further constrained by colonization resistance driven by host-associated microbiota and immune factors, which can prevent their interaction with epithelial or immune cells (42). Moreover, in the context of epithelial barrier dysfunction, probiotics are unable to effectively reinforce barrier integrity or induce tolerogenic signaling, limiting their capacity to suppress pro-inflammatory pathways (43). Therefore, large-scale multicenter RCTs are needed to better assess the role of probiotics in asthma management and address the uncertainties raised by TSA analysis.

Marked heterogeneity was detected across several outcomes (*I*^2^ > 80%), prompting the use of a random-effects model. This inconsistency likely reflects substantial clinical variation in probiotic interventions, including differences in strain composition (single-strain versus multi-strain), dosage (3.1 × 10 to 10 × 10 CFU/day), and treatment duration (2–12 weeks), as well as variations in participant characteristics such as age (ranging from 1 to 18 years) and the presence of comorbid atopic conditions. Given that most of the outcomes considered were reported in only two or three trials, subgroup analyses to further explore the sources of heterogeneity were not feasible. Consequently, the high heterogeneity observed in this study limits the robustness and generalizability of the findings. To address this issue, future research should include large-scale RCTs with standardized probiotic strains, dosages, and treatment durations, clearly defined participant characteristics, and consistent outcome measures to reduce clinical and methodological heterogeneity.

Regarding safety, only Ding et al. (27) and Chen et al. (23) explicitly reported no adverse events related to the administration of probiotics to pediatric patients with asthma, whereas other studies did not mention any safety outcomes. Therefore, we did not perform a meta-analysis on safety data. Although Hassanzad et al. (44) reported one case of vomiting leading to treatment withdrawal among 51 pediatric patients with asthma receiving synbiotic treatment, this isolated finding does not substantially strengthen the evidence for potential safety concerns. Moreover, the incomplete reporting of adverse events and the short duration of the interventions in the included trials further constrain the ability to detect rare or delayed probiotic-related complications. Taken together, the available evidence is insufficient to draw reliable conclusions regarding the safety of probiotics in in pediatric patients with asthma, and the findings should be interpreted with caution. Larger, well-designed, multicenter RCTs with standardized and comprehensive adverse event monitoring are required to provide a more definitive assessment of the safety profile of probiotics in this patient subpopulation.

This meta-analysis has several limitations. First, the diagnostic criteria used across the included studies varied, encompassing the Global Initiative for Asthma guidelines, the 2016 Guidelines for the Diagnosis and Prevention of Childhood Bronchial Asthma (45), and the 2008 Guidelines for the Diagnosis and Prevention of Childhood Bronchial Asthma (46), which may have introduced clinical heterogeneity to the analysis. Second, only six clinical trials involving a total of 731 patients were included, which may have reduced the precision and statistical power of the pooled estimates. Third, all studies were conducted in China. This geographic concentration substantially limits the external validity of the findings, as the results primarily reflect outcomes in Chinese pediatric patients with asthma and may not necessarily be applicable to patients in other regions, healthcare systems, or ethnic groups. Fourth, the intervention duration in the included studies ranged from 2 to 12 weeks, and therefore the long-term effects of probiotics on pediatric asthma could not be properly analyzed. Considering these limitations, further research is warranted. Future studies should be designed as large-scale, multicenter, double-blind RCTs with a particular focus on evaluating the effects of probiotics on airway inflammation and pulmonary function to provide stronger evidence for their clinical application in pediatric asthma. In addition, stratified analyses based on probiotics strains, dosage, and treatment durations are needed to establish standardized therapeutic protocols for probiotics use in pediatric patients with asthma.

## 5 Conclusion

Although probiotics appeared to influence IL-4 and IFN-γ levels, the very low certainty of the evidence, inconclusive TSA results, and the fact that all included RCTs were conducted in China limit the external validity and clinical reliability of the findings of this meta-analysis. Furthermore, the incomplete reporting of safety outcomes prevents a definitive assessment of the safety profile of probiotics in pediatric patients with asthma. Therefore, the available evidence does not support the routine use of probiotics as adjunctive therapy in these patients. Large-scale, multicenter, double-blind, RCTs are needed to clarify the potential role of probiotics in asthma management and provide more robust evidence for their clinical application.

## Data Availability

The original contributions presented in the study are included in the article/Supplementary material, further inquiries can be directe d to the corresponding authors.
